# Biomimetic light dilution using side-emitting optical fiber for enhancing the productivity of microalgae reactors

**DOI:** 10.1038/s41598-019-45955-w

**Published:** 2019-07-03

**Authors:** Lothar Wondraczek, Alexander Gründler, Aaron Reupert, Katrin Wondraczek, Markus A. Schmidt, Georg Pohnert, Stefan Nolte

**Affiliations:** 1 Otto Schott Institute of Materials Research, University of Jena, Fraunhoferstrasse 6, 07743 Jena, Germany; 2 Abbe Center of Photonics, University of Jena, Albert-Einstein-Strasse 6, 07745 Jena, Germany; 3 Center of Energy and Environmental Chemistry, University of Jena, Philosophenweg 7, 07743 Jena, Germany; 4 Leibniz Institute of Photonic Technology, Albert-Einstein-Strasse 9, 07745 Jena, Germany; 5 Institute of General and Inorganic Chemistry, University of Jena, Humboldtstrasse 8, 07745 Jena, Germany; 6 Max Planck Institute for Chemical Ecology, Hans-Knöll-Strasse 8, 07745 Jena, Germany; 7 Institute of Applied Physics, University of Jena, Albert-Einstein-Strasse 15, 07745 Jena, Germany

**Keywords:** Applied optics, Materials for energy and catalysis, Chemical engineering, Materials for optics, Fossil fuels

## Abstract

Photoautotrophic microbes present vast opportunities for sustainable lipid production, CO_2_ storage and green chemistry, for example, using microalgae beds to generate biofuels. A major challenge of microalgae cultivation and other photochemical reactors is the efficiency of light delivery. In order to break even on large scale, dedicated photon management will be required across all levels of reactor hierarchy – from the harvesting of light and its efficient injection and distribution inside of the reactor to the design of optical antenna and pathways of energy transfer on molecular scale. Here, we discuss a biomimetic approach for light dilution which enables homogeneous illumination of large reactor volumes with high optical density. We show that the immersion of side-emitting optical fiber within the reactor can enhance the fraction of illuminated volume by more than two orders of magnitude already at cell densities as low as ~5 10^4^ ml^−1^. Using the green algae *Haematococcus pluvialis* as a model system, we demonstrate an increase in the rate of reproduction by up to 93%. Beyond micoralgae, the versatile properties of side-emitting fiber enable the injection and dilution of light with tailored spectral and temporal characteristics into virtually any reactor containment.

## Introduction

The aquatic biosphere accounts for about half of the global carbon fixation^1^, using photosynthesis to store solar energy and CO_2_ in complex chemical compounds. Harnessing microbial beds such as in microalgae reactors presents obvious opportunities for sustainable energy conversion, CO_2_ storage and the production of fine chemicals^2,3^. For example, significant progress in genetic engineering, lipid harvesting and refining led to predictions which foresee large-scale production of biodiesel from microalgae within the next decade^4^. Even in direct comparison to photovoltaic technology with notoriously higher conversion efficiency^5^, microbial photosynthesis holds the promise of broadest versatility and remains unchallenged in the production of high-value products by green chemistry^6–10^. Therefore, while various technical issues seem to be delaying the original roadmap at least for biofuels, the prospect of microalgae technology remains unabated^11^.

A major challenge in aquatic photosynthesis and, in fact, all photochemical reaction beds is the efficient delivery of light to the photochemical machinery. This starts with the light source (and, in case of solar illumination, the choice of location^12^) and encompasses all subsequent optical interfaces down to intracellular antenna designs (*e.g*.^13^). Realistic estimates of the theoretical productivity of microalgae reactors predict an annual output of unrefined oil of ~35 L.m^−2^ ^14^. In real world, best-case scenarios reach roughly one tenth of this value. Aside of the intrinsic efficiency of the photosynthetic machinery, the primary factors which contribute to this discrepancy are all connected to illumination efficiency: spectral loss occurs due to the spectral mismatch between the incoming radiation and the sensitivity curve of the photosynthetic dye^15^, transmission loss is caused by reflecting surfaces, interlayers or containments^16^, and photon utilization loss results from quenching effects such as photo inhibition, heat generation, or a mismatch between photon energy and dye band-gap^14,17^. On this line, reactor layout and implementation remain the most important factors for economic success^18^: enhancing the productivity of microalgae cultivation systems will require dedicated optical engineering and photon management on reactor level^19–22^. The key question is: How can light with appropriate spectral and temporal characteristics be delivered to the darkest regions of a reactor containment in the most efficient and homogeneous way? This general subject encompasses many similar problems as they occur in different applications or on different length scale (e.g., illumination of soil^23^ or artificial microbial arenas^24^).

Microalgae cultivation demands illumination schemes with spectrally tailored intensity and complex temporal cycling^16^. Starting from the threshold of minimum light intensity, the production rate of a photosynthetic apparatus is usually increasing with increasing irradiance up to the onset of photo inhibition with an empirical threshold in photon flux density (PFD, integrating over the spectral range of 400–700 nm) of 200–400 µmol m^−2^ s^−1^ ^17^. Depending on algae species, typically employed illumination densities range within 50–200 µmol.m^−2^ s^−1^ ^25^. These are applied in tailored light-dark cycles which are facilitated by mixing operations^26,27^. Reactors themselves are implemented in the form of open ponds, km-long glass or plastic tubings, or flat panel devices^28^.

**Figure 1:**
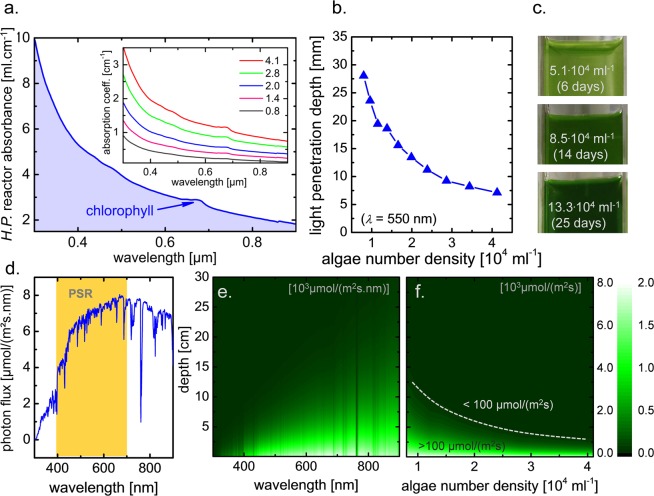
Optical attenuation and light penetration in a microalgae bed. (**a**) Spectral distribution of the direct molar absorbance of a reactor filled with H. pluvialis within the visible spectral domain. The arrow highlights the absorption of chlorophyll-a at around 680 nm. The inset shows examples of the wavelength dependence of the absorption coefficient for different algae concentrations (labels indicate five selected concentrations in units of 10^4^ ml^−1^). (**b**) Light penetration depth as a function of algae number density. Representative photographs are provided in (**c**) for different concentrations and days of cultivation, respectively (the depicted container width is 35 mm). (**d**) Spectral photon flux density for solar illumination at air mass (AM) 1.5 (irradiance of 1000 W/m^2^), calculated from ASTM G173-03 standard tables. The highlighted region corresponds to the photosynthetic range (PSR). (**e**) Spectral photon flux density inside the reactor for 100% solar injection (AM1.5, taken from panel (d)). (**f**) Total photon flux covering the spectral range of 400-700 nm (PSR) as a function of reactor depth and algae concentration. The dashed line in (**f**) marks an empirical threshold between typical reactor operation and oversaturation.

Delivery of light into the deeper regions of microalgae reactors forces a trade-off between the volume density of microbial species and the illumination efficiency: the depth to which incoming light penetrates into the reactor is reduced by scattering and absorption. As a result, for a certain local illumination intensity, limits are imposed on microbe number density, pigment concentration and reactor geometry. As an example, for a reactor containing the green microalgae *Hematococcus pluvialis*, the relative light transmission of the algae suspension decreases exponentially with increasing algae number density (Fig. 1a). Even for concentrations which approach the lower limit of being technologically acceptable in terms of reactor throughput, the light penetration depth decreases rapidly to less than 10 mm (Fig. 1b). In present reactor technology, the batch depth ranges from a few centimeters (flat panel and tubular reactors) to ~40 cm (open pond reactors)^29^. Optical attenuation leads to strongly heterogeneous light distribution, with large oversaturation in the outer regions of the reactor, and literal darkness inside. Dedicated liquid mixing and agitation strategies may only partially overcome this issue. On the promising side, it was suggested that the productivity of microalgae reactors could be enhanced about fourfold by ideal, volumetric light dilution^30^.

The outlined problem applies to basically any photochemical machinery. Alternative schemes of illumination which enable volumetric, homogeneous light delivery throughout the reactor containment are of strong interest^31^. In comparison to illumination from an outer surface, immersing the light source inside the reactor enhances the individual proximity of illumination and, thus, may largely overcome the limitations of optical attenuation. As an additional benefit, such systems allow for the separation of light generation or collection and algae cultivation by using fiber optical waveguides^32–34^. Besides a significant reduction of heat load, this provides access to the whole toolbox of spectral engineering, including intensity modulation, temporal cycling and spectral conversion. Various designs have therefore been tested for implementing volumetric light dilution, for example, using light-guides with diffusing surfaces such as plates^35^, tubes^36^, rods^37^ or fiber^38–42^. In particular the latter seems to be most promising due to the following reasons: First, highest flexibility is ensured in terms of geometrical arrangement into a skeleton of light strands with adapted illumination density and microfluidic properties. Secondly, a very large effective illumination area is achieved at the fiber-liquid interface when using a lattice of multiple light-emitting fibers.

## Results

### Biomimetic light delivery

**Figure 2:**
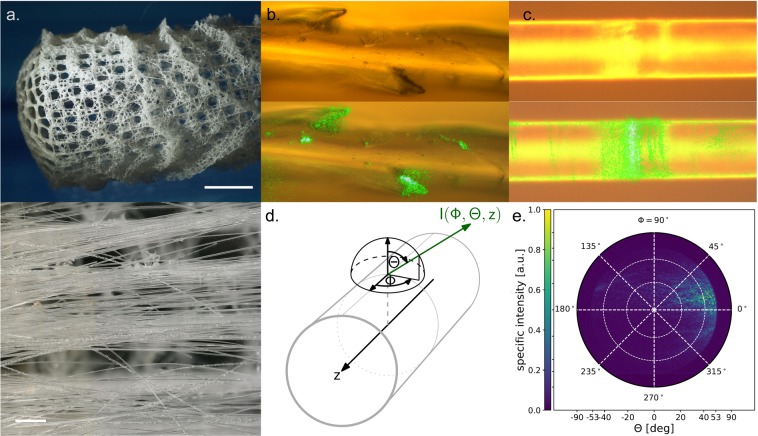
Light transport and side-emission in the deep sea sponge *E. Aspergillum*. (**a**) Overview photographs of *s* in the osculum (top, scale bar 1 cm) and basal (bottom, scale bar 1 mm) region. (**b**) Detailed view at a spicule with pronounced spines (top) and green light emission from the spine region (bottom). (**c**) Detailed view at a homogeneous section of E. aspergillum spicule fiber (top) and green light emission being concentrated at some interior scattering center (bottom). (**d**) Schematic of the angular distribution of light emission and (**e**) pole figure of light emission, recorded by Fourier microscopy on the E. aspergillum spicule (objective NA = 0.8) shown in panel (**c**). Bottom figures in (**b**) and (**c**) are overlays.

A lesson on volumetric light dilution can be learnt from nature by looking at the opposite problem of systems which efficiently harvest light from environments with low illumination intensity. Very prominent examples for this are siliceous sponges such as the archetype hexactinellid species of *Euplectella aspergillum*^43,44^. *E. aspergillum* comprises of a skeleton of basalia spicules (Fig. 2a). These biological silica fibers are structured across multiple levels of hierarchy down to nanometer-scale silica spheres which are packed in a way to allow for multi-mode light guidance^38^. Interestingly, on the higher levels of hierarchy, the spicules exhibit a refractive index which is decreasing from core to surface. This allows for a reduction of surface reflectivity in aqueous environment and enhances the ability of light harvesting^16^. In addition, lens-like structures at the fiber ends and individual spines on their surface have been identified as illumination points which further enhance the efficiency of light harvesting (see also Fig. 2b). Interestingly, in the same way in which this design allows for light harvesting, it also enables volumetric illumination when light is fed into the fiber ends, distributed throughout the sponge, and reemitted from the spine surfaces. This is illustrated in Fig. 2b–e, using Fourier microscopy to quantitatively image the angular distribution of light emission. The light-guiding spicules have a diameter of ~50 µm (Fig. 2c) and an average refractive index of ~1.43^38^. In water with *n* = 1.33, this corresponds to a numerical aperture of *NA*~0.53, which is similar to that of a typical PMMA fiber (*NA*~0.5). Light emission from the spicules occurs predominantly through scattering at the spines (Fig. 2b) or at interior imperfections (Fig. 2c). Due to the surface gradient in refractive index^38^, furthermore, a pronounced evanescent field surrounds the major part of the spicule surface.

The angular characteristics of the light scattering behavior of *E. aspergillum* spicules and side-emitting fiber were obtained by Fourier microscopy^45^. As shown in Fig. 2e the *E. aspergillum* spicule fiber exhibits strong scattering at low angles (*θ* < 40°) with respect to the direction of light guidance. This indicates that the refractive index fluctuations which are responsible for the observed light emission have a larger spatial frequency than the frequency of the scattered light^46^. From the absence of backscattering we can conclude that Rayleigh scattering (caused by fluctuations of higher special frequency than visible light) plays only a minor role in the present scattering process.

### Illumination performance analysis

**Figure 3:**
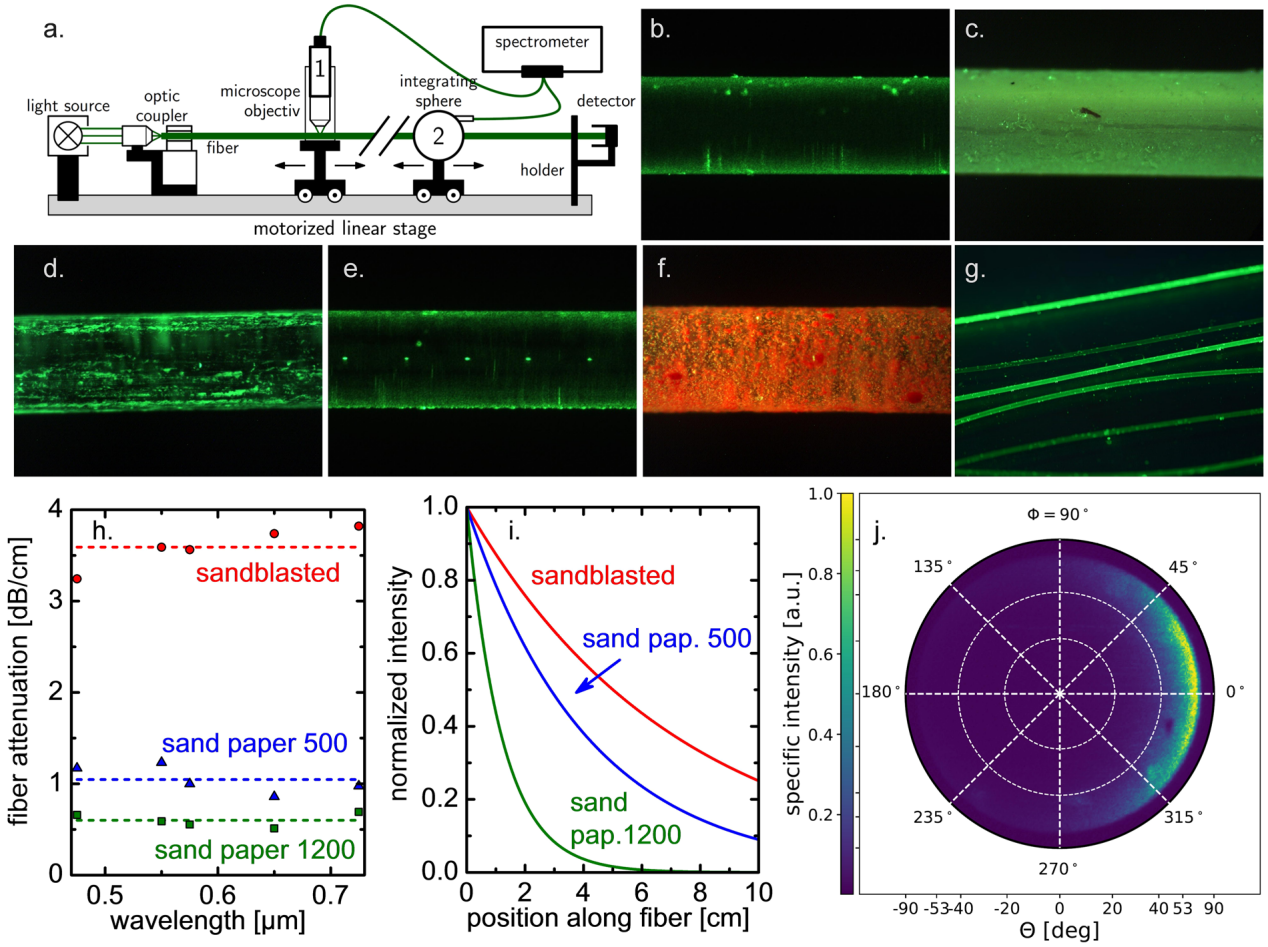
Side-emitting fiber for volumetric illumination of aqueous suspensions. (**a**) Experimental set-up for light injection and analysis of emission characteristics. (**b**–**f**) Examples of light-emitting fiber types: bare PMMA fiber with intrinsic turbidity (**b**), PMMA fiber after etching in acetone (**c**), PMMA fiber after scratching on sand paper (**d**), silica fiber with laser-writing grating (**e**), PMMA fiber with CaSrS:Eu^2+^/epoxy coating (**f**). Photographs (**b**–**f**) were taken in air. In (**g**), a PMMA fiber is immersed in water. Plastic fibers in (**b**-**g**) have a diameter of d = 750 µm. The silica fiber in (**e**) has d = 400 µm. For the photographs, light injection was done with a green cw laser at 100 mW. Panels (**h**–**j**) present emission properties of selected PMMA fibers obtained in air. In (**h**) the spectral dependence of optical attenuation is shown for fiber sections of ~50 cm, using mechanically roughened plastic fiber as indicated by the labels. The corresponding normalized intensity of side-emission is shown in panel (i). (**e**) Pole figure of light emission, recorded by Fourier microscopy on the plastic fiber shown in panel (**g**), using an objective NA of 0.8.

Side-emitting fiber mimics the light emission behavior of *E. aspergillum* spicules. Through post-processing (or through variations on the fiber drawing process or the preform itself), the emission behavior can be tailored in terms of spectral, lateral and angular characteristics. In Fig. 3, we display some examples which illustrate this high versatility, including bare plastic fiber with inherent turbidity (Fig. 3b), plastic fiber with a chemically treated surface (Fig. 3c), surface roughening on plastic fiber (Fig. 3d), laser-written gratings in silica glass fiber (Fig. 3e) and the application of a coating comprising a light-converting dye (Fig. 3f^15^). In all approaches light scattering refractive index fluctuations are generated in the fiber or at its surface, promoting light extraction from the guiding core. In principle, any of these variants can be employed in the form of centimeter- to meter-long strands for immersed illumination of aqueous environments (Fig. 3g). For practicality, the ability of disinfection/autoclave treatment prior to experiments and the persistence of the scattering effect when immersed in a liquid need to be considered in the specific case.

For a constant scattering function, the fraction of guided light within the core decreases exponentially (Fig. 3h). In parallel, also the intensity of light emission from the fiber surface decreases along the length of the fiber (Fig. 3i). Outside of the scope of the present report, the generation of homogeneous side-emission from the fiber presents a remaining challenge for which several solutions have been proposed^47,48^.

For simplicity, plastic fiber similar to Fig. 3d,h was employed in the following. Depending on surface roughness, they exhibited an optical attenuation of ~0.5 dB/cm up to 3.5 dB/cm. In the demonstration case, the length of the light-diffusing fiber section is 10 cm. In order to attenuate 99% of the injected light through scattering (using air-emission for reference), we target for ~2 dB/cm (Fig. 3h, further details are provided in the *Materials and Methods* section). The angular emission characteristics of the plastic reference fiber are very similar to those which have been observed on the *E. aspergillum* spicule (Fig. 3j). Pronounced low angle emission and the absence of backscattering confirm that the refractive index fluctuations occur on larger special frequency than the scattered light. In contrast to the angular scattering pattern of the *E. aspergillum* which also exhibits scattering components at larger angle, the Fourier map of the technical fiber is relatively homogeneous. The larger-angle reflexes visible on *E. aspergillum* are linked to special frequencies similar to the scattered light caused by finer structures and/or sharper refractive index transitions such as cracks, pores or similar defects.

**Figure 4:**
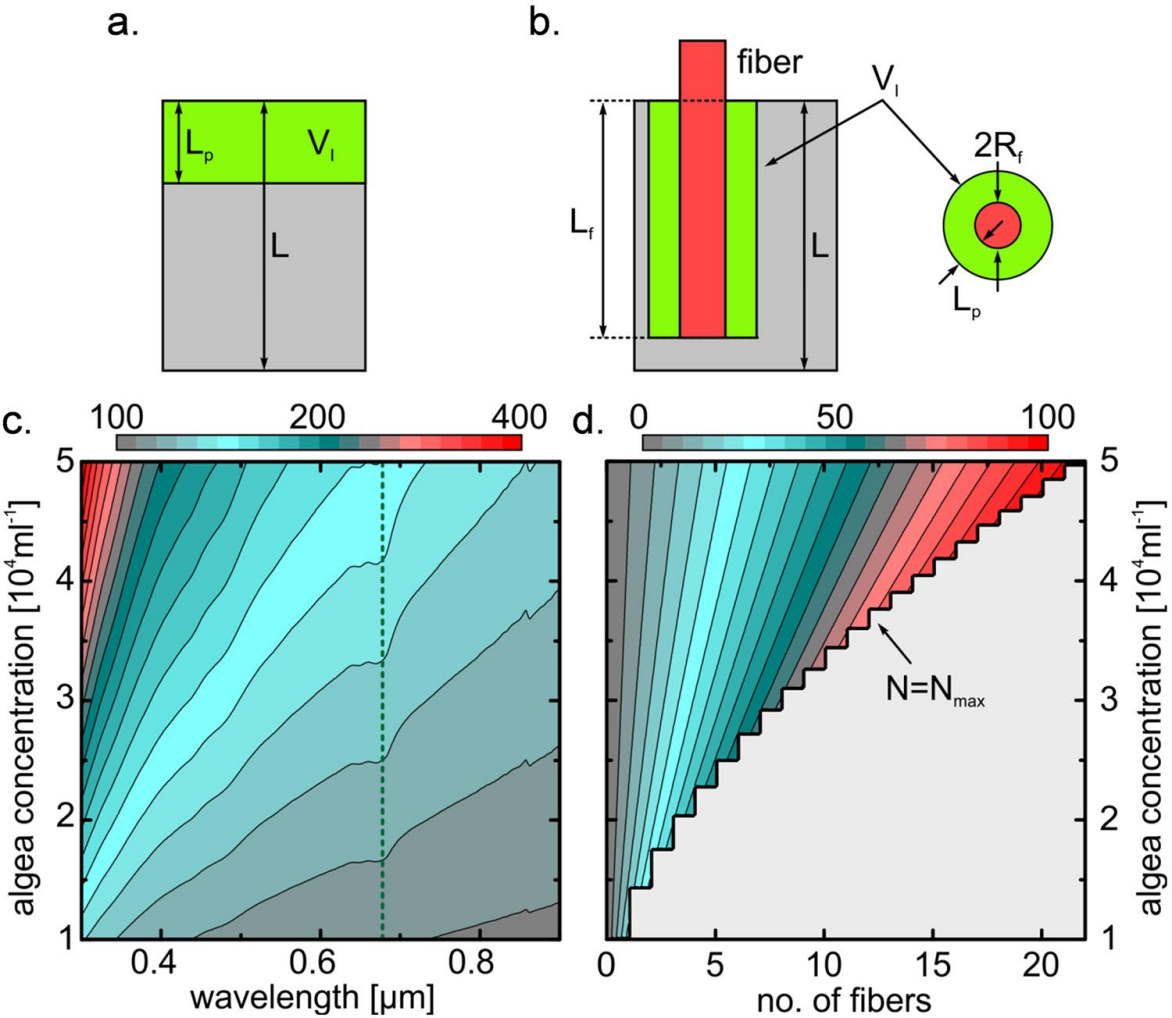
Performance analysis of the fiber-enhanced algae reactor by comparing the illuminated volumes of a bare reactor illuminated from the top without fibers and a similar reactor containing the side-emitting fibers. The sketches in the upper row illustrate the two types of reactor ((**a**) bare reactor, (**b**) fiber-enhanced reactor) with gray representing the reactor volume, green the light penetration depth into the algae solution (and thus the illuminated volume) and red the fiber. (**c**) Contour plot of the enhancement factor (per unit length) as a function of algae concentration and wavelength (value of ${K}_{n}^{\max }$ is represented by the top color bar) when the maximum number of fibers are used (N = N_max_, R_f_ = 500 µm). The vertical green dashed line indicates the chlorophyll absorption band at ~680 nm. (**d**) Example of the dependence of the enhancement factor on algae concentration and number of fiber located inside the reactor (top color bar refers to the values of K_n_) at the chlorophyll absorption wavelength (indicated by the vertical green line in (**c**)), calculated for a predefined reactor configuration. The gray area refers to the impractical situation of N > N_max_.

We now consider the theoretical extent to which local illumination can be improved through the discussed approach. For this, we compare a conventional set-up in which a reactor containment is illuminated from the top (Fig. 4a) and the use of side-emitting fiber immersed inside the reactor (Fig. 4b). The illuminated volume *V*_I_ results from the area of illumination *A* and light penetration depth into the algae solution *L*_*p*_, ${V}_{I}=A{L}_{p}$. We define the penetration depth as the maximum distance (for emission perpendicular to the fiber axis) were the light transmission has dropped to 1/e, so according to the Lambert-Beer’s law.

${L}_{p}={(\varepsilon c)}^{-1}$, with the specific suspension absorbance *ε* and the concentration of the microalgae *c*. For simplicity, this treats the microalgae as homogeneous absorbers and the light as being collimated. Then, *ε* is a sum of contributions from absorbance, scattering, shadow effects, etc. Light dilution is achieved by immersing multiple fibers within the sample volume, each representing an individual strand of light. Obviously, the efficiency of light dilution depends on the specific arrangement of the fiber lattice, for example, in simple square arrangement, more dense hexagonal arrangement, or even in 3-dimensional geometries such as spirals or wood pile designs. For the simple case of an ensemble of *N* fibers in a uniform square lattice, with the immersed length *L*_f_ and radius *R*_f_ it is

1 $${V}_{I}=\pi N{L}_{f}[{({R}_{f}+{L}_{p})}^{2}-{R}_{f}^{2}]$$

The maximum number of fibers *N*_max_ is estimated by

2 $${N}_{{\rm{\max }}}=\,{\rm{int}}[A/(4{({R}_{f}+{L}_{p})}^{2})]$$

where the int-function is required for obtaining discrete values of *N*_max_, leading to the step-wise behavior displayed in Fig. 2d. The value of *N*_max_ signifies the limit at which the illuminated volumes which surround each fiber start to overlap. Constraints of this model are that $N\le {N}_{\max }$ and ${L}_{f}\le L$. The extent to which the use of immersed fibers increases the illuminated volume can be described through an enhancement factor, ${K}_{n}=\pi N{L}_{f}[{({R}_{f}+{L}_{p})}^{2}-{R}_{f}^{2}]/(A{L}_{p})$. The maximum enhancement factor per unit length (with the maximum possible number of fibers and with *L*_*f*_ = *L*) is given by

3 $${K}_{n}^{{\rm{\max }}}=\frac{\pi }{4}\frac{2{R}_{f}+{L}_{p}}{{({R}_{f}+{L}_{p})}^{2}}$$

If the radius of the employed fibers is notably smaller than the light penetration depth, *R*_*f*_ ≪*L*_*p*_, the maximum enhancement factor per unit length can be simplified to

4 $${K}_{n}^{{\rm{\max }}}\approx \frac{\pi }{4{L}_{p}}$$

This latter condition is valid when light-guiding optical fiber with typical radii *R*_*f*_ < 500 µm are employed relative to *L*_p_ > 5 mm (Fig. 1). Accordingly, higher enhancement factors are achieved in suspension with higher number density of algae, and thus the fiber-based approach is particularly useful for illuminating optically dense suspensions. Overall, this analysis shows that an enhancement in the illuminated volume of one to two orders of magnitude can readily be achieved, even when *N* < *N*_max_ (what may be dictated by rheological and micro-fluidic considerations, outside the present scope). As will be demonstrated in the following, in a true cultivation experiment, the number density of microbes is not a constant but increasing gradually with prolonged time of cultivation. Therefore, *L*_p_ is decreasing and hence, fiber illumination becomes gradually more efficient relative of conventional illumination.

Noteworthy, this evaluation provides a simple metric for judging the relative enhancement of the volume which receives at least 1/e of the light intensity which was initially injected at the fiber-suspension interface. It does not take into account the further fact that also within this illuminated volume, the local intensity varies. Since the sensitivity of the respective photochemical machinery may depend on intensity, quantitative conclusions on the actual enhancement of system productivity require knowledge of this sensitivity function.

### Enhancement of photosynthetic activity and reactor performance

**Figure 5:**
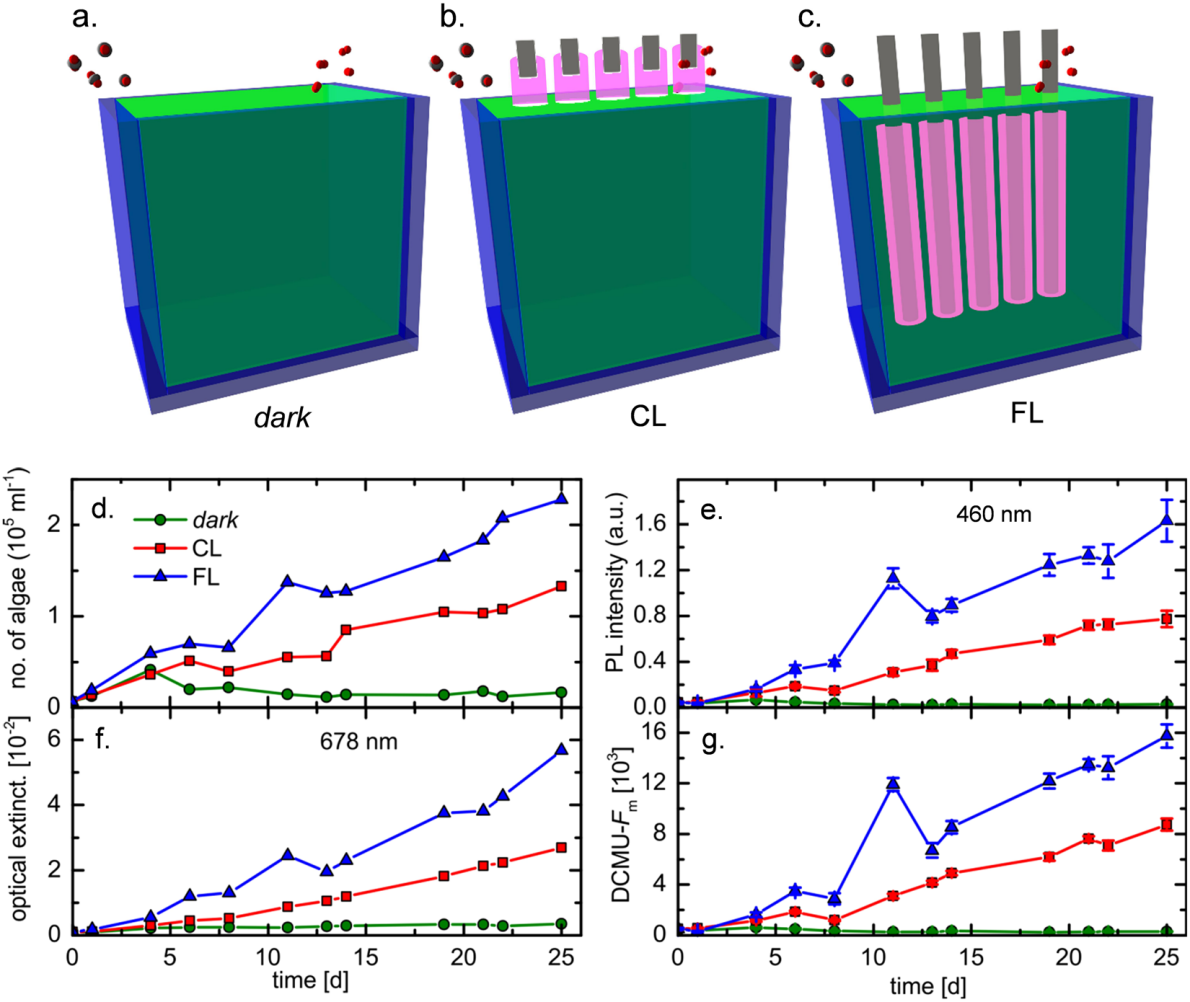
Productivity of H. pluvialis microalgae reactor for varying illumination methods. Panels (a–c) show the different reactor set-ups, without illumination (dark, (**a**)), with conventional illumination from the top of the algae bed (CL, (**b**)) and with illumination through immersed side-emitting fiber (FL, (**c**)). The corresponding reactor productivity is shown as a function of cultivation progress in panels (d–g), monitoring algae number density (**d**), intensity of absolute chlorophyll-a photoluminescence (**e**), optical density of the reactor (**f**) and chlorophyll-a photoluminescence after DCMU treatment (**g**).

At an initial microalgae cell density of ~0.7 · 10^4^ ml^−1^ the light penetration depth into the reactor is in the range of 50 mm (Fig. 1b). As a consequence, there is only little difference in the illuminated volume between conventional illumination from the top-surface (CL) and fiber-assisted illumination (FL) configurations: for a reactor size such as employed in the present demonstration experiment with *L* = 95 mm, top-face illumination reaches *I/I*_0_ > 1/e in more than 50% of the total reactor volume. The remaining reactor volume is still illuminated at an intensity level lower than 37% of the incoming irradiation, decaying to ~15% at the bottom of the reactor. We see a practical threshold at which fiber illumination becomes efficient when the algae cell density approaches ~5·10^4^ ml^−1^ (*L*_p_ < 5 mm, *V*_I_/*V*_tot_^CL^~5%). At this value, the volume fraction which can be illuminated in CL configuration becomes so small that switching to FL configuration appears advantageous. This is confirmed in the experimental data shown in Fig. 5d–g. Over the course of 25 days of cultivation, algae cell counts, chlorophyll-a concentration, and photosynthetic activity increase relative to the dark control sample, both in CL and FL configuration. A notable effect of the illumination method starts to set-in after about 5 days of cultivation, when a threshold in microbe density is reached at around 5·10^4^ ml^−1^. From there onwards, the continuous increase in microalgae density (accompanied by rapidly decreasing *L*_p_) leads to a pronounced effect of volumetric illumination in FL configuration, i.e., with up to 93% (22 days) enhancement in absolute algae number density as compared to CL illumination. At the same time, the chloroplast photoluminescence intensity is enhanced by ~76%, and DCMU-*F*_m_ by 87%.

## Discussion

Efficient light delivery presents one of the major obstacles in large-scale exploitation of microalgae reactors. This is due to strong light extinction at elevated pigment and cell densities which can reduce the light penetration depth of reactor beds to a few millimeters. Then, conventional illumination from external light sources may cause strong oversaturation or even photoinhibition at the reactor surface while the interior of the reactor remains in the dark. Side-emitting fiber provides a biomimetic approach for light dilution and homogeneous illumination of large reactor volumes with high optical density. In such light guides, lateral light emission is obtained through introducing a longitudinal scattering function. With fiber designs reaching from materials with inherent turbidity and facile procedures for applying surface roughness through grinding, scratching or chemical treatments to laser-written scattering centers or the application of functional coatings, the temporal, spectral and spatial characteristics of light emission can be broadly tailored. This facilitates the adaption to specific microbial environments, for example, through providing spectrally selective light or light/dark cycles. While homogeneous side-emission, fiber cleansing and disinfection, surface algal growth and fouling present remaining challenges, solutions which implement secondary surface modifications such as laterally decaying scattering functions and repellent layers are anticipated.

A performance analysis revealed that the fraction of illuminated volume of a given reactor can readily be enhanced by more than two orders of magnitude in comparison to conventional illumination when using side-emitting fiber for spatially diluted illumination. Such an enhancement can be acieved even at comparably low cell densities, *e.g*., as low as ~5   10^4^ ml^−1^ for a reactor filled with the green algae *H. pluvialis*. Using *H. pluvialis* as a model system, we demonstrated an increase in the rate of reproduction by up to 93%, and of chlorophyll-a activity by about three thirds.

## Materials and Methods

### Sample preparation and side-emission

Fiber and spicule characterization involved different microscopic techniques, including surface roughness studies by wide-field confocal microscopy (Zeiss Smartproof 5, Germany), digital imaging (Keyence VHX 6000, Japan), and polarization microscopy (Zeiss JENAPOL, Germany). The angular side-emission behavior of *E. aspergillum* spicules and of light-guiding fibers was analyzed through Fourier microscopy by inserting a Bertrand lens into the light path of the polarization microscope. Fourier microscopy uses the property of lenses to decompose the radiation field in its front focal plane into its angular components in its back focal plane, referred to as the Fourier transform property of lenses. The result of this measurement is a pole figure, as show in Figs 2e and 3j, which can be understood as the hemisphere in Fig. 2d when looked at from the top: every ray emerging from the center of the sphere, which coincides with a surface element of the fiber in the focal point of the lens, pierces the sphere at a certain position corresponding to the angles *ϕ* and *θ*. The specific intensity in this direction is indicated by the color scale the data plot. The maximum angle *θ* which can be measured with this arrangement is determined by the numerical aperture of the lens, *NA* = sin θ (in air). The full set-up of light injection and characterization of the emission characteristics is shown schematically in Fig. 3a, whereby Fourier microcopy and intensity analyses were conducted separately. As light source, these experiments used a cw laser source at varying frequency (100 mW). Quantitative emission intensity analyses were performed using the motorized scanning stage shown in Fig. 3a equipped with an custom-built integration sphere (PTFE) and a spectrometer (Maya2000 Pro). Measurements of the optical loss within the fiber were performed by the cut-back method, detecting transmitted intensity as a function of fiber length.

### Microalgae cultivation and suspension analyses

*Haematococcus pluvialis* microalgae (strain no. 192.80, purchased from the Culture Collection of Algae at Goettingen University, SAG, Germany, in 2014) were used as a model species for demonstrating reactor efficiency. *H. pluvialis* is a green freshwater microalgae. In its non-motile state, it produces the antioxidant carotenoid dye astaxanthin, with numerous applications in nutrition, cosmetics and health^49,50^. Together with its relatively high resilience, this technological importance made it a widely-employed model for laboratory studies, including reactor design and performance analyses (e.g.^19^). Starting with 100 ml of suspension volume, initial cultivation was conducted in Bold’s basal medium plus vitamins (BBM-V) in order to provide sufficient material for reactor tests. BBM-V was produced according to established protocols^51,52^. Cultivation was performed in transparent polycarbonate bottles of 600 ml over the course of 20 days (doubling the suspension volume after 15 days), at 18 °C with a simulated day/night cycle of 14 h of illumination (10–28 µE) followed by 10 h of darkness. At this stage, a biomass of (1.31 ± 0.01) g of dry algae per kg of suspension was obtained according to gravimetric analysis. After initial cultivation, 1 L of BBM-V was added to 200 ml of algae suspension. From this mixture, 360 ml each were filled into three test reactors, to be used in dark, CL and FL configuration, respectively. In order to quantify the effects of different illumination configurations, microbe number density and photosynthetic activity were determined as a function of cultivation time by extracting samples of 500 µl of suspension after specified time intervals of cultivation (typically one day). If not stated otherwise, Lugol’s solution (15 µl) was used for sample fixation. The microalgae density was determined directly through cell counting, and indirectly through measuring the optical density of such samples. Cell counting was conducted in Fuchs-Rosenthal counting chambers with a cell depth of 0.2 mm and a grid spacing of 0.25 mm, using a total volume of 3.2–6.4 µl (256–512 wells). Across all experiments, the absolute number of counted algae was within 40–650. The initial algae cell density in the suspension determined in this way was ~0.692 10^4^ ml^−1^.

The optical extinction was recorded on a UV-Vis spectrophotometer (Shimadzu UV-3101) over the spectral range of 300–900 nm, using suspension samples in 9.98 mm × 9.98 mm silica cuvettes. The optical extinction value used in Fig. 5 was extracted from the absorbance at 678 nm (for a path length of 9.98 mm). The photosynthetic activity was subsequently judged on the basis of chlorophyll-a photoluminescence. For this, fluorescence spectroscopy was conducted (Mithras Lb 940, using a 75 W halogen lamp for excitation) following the protocol of Maxwell and Johnson^53^. The wavelength of 430 nm was selected for excitation, matching the excitation band maximum of chlorophyll-a in *H. pluvialis*^19^. The emission intensity was recorded at a wavelength 460 nm. Samples were prepared on 96-well plates with 0.2 ml individual volume. Measurements were conducted directly after sampling, 0.5 h after dark storage, and after addition of 15 µl of the algicide 3-(3,4-dichlorphenyl)-1,1-dimethylurea (DCMU). DCMU inhibits PSII, hence providing the maximum fluorescence level *F*_m_.

If not stated otherwise, all devices and tools used in the preparation and handling of algae suspensions were sterilized in an autoclave at 120 °C for 1 h prior to use.

### Reactor set-up

The test reactors had a cross-section of 35 mm × 108 mm (*A* = 3780 mm²) and a rectangular height of 130 mm. Filled at 360 mL, this corresponds to a reactor depth *L*~95 mm. The three reactor set-ups which were run in parallel are depicted schematically in Fig. 5a–c.

For reactor illumination, the demonstration experiments used plastic light guiding fiber pieces (PMMA) with a diameter of 750 µm a length of 35 cm. A homogeneous surface roughness was generated on the last ~10 cm of fiber surface through mild grinding on 240 grit silicon carbide sandpaper (see Fig. 3d). From the roughened fiber, bundles of 400 pieces with a length of 30–45 cm were assembled for insertion into the reactor devices as depicted in Fig. 5c. The bundles were fixed on one end (non-roughened side) with a 2 cm shrink hose, and polished. Light was fed into the bundle from the fixed side, using a 15 W RGB LED generator (Stiers, Germany). The injected photon flux from the sum of all elements was ~20 µmol m^−2^ s^−1^. In CL configuration, a similar fiber bundle was put on top of the microalgae suspension without immersion; in FL configuration, the bundle was placed so that the roughened parts of the fibers were immersed in the liquid. Before application in algae suspensions, fiber bundles were sterilized. Cultivation experiments were performed over the course of 25 days, running all three reactors in parallel, and repeated once.

## Data Availability

The datasets generated and/or analyzed during the current study are available from the corresponding author on reasonable request.
